# Isocitrate Dehydrogenase Mutations in Glioma: From Basic Discovery to Therapeutics Development

**DOI:** 10.3389/fonc.2019.00506

**Published:** 2019-06-12

**Authors:** Juan Huang, Jialong Yu, Lin Tu, Nanqu Huang, Hang Li, Yong Luo

**Affiliations:** ^1^Key Laboratory of Basic Pharmacology and Joint International Research Laboratory of Ethnomedicine of Ministry of Education, Zunyi Medical University, Guizhou, China; ^2^The Third Affiliated Hospital of Zunyi Medical University, The First People's Hospital of Zunyi, Guizhou, China

**Keywords:** isocitrate dehydrogenase, glioma, IDH inhibitor, IDH vaccine, 2-hydroxyglutaric acid

## Abstract

Isocitrate dehydrogenase (IDH) is a key rate-limiting enzyme in the Krebs cycle that plays an important role in energy metabolism. In recent years, it has been found that IDH mutations are closely related to the occurrence and development of glioma, and it is a notable potential therapeutic target. First, IDH mutations can produce high levels of 2-hydroxyglutaric acid (2-HG), thereby inhibiting glioma stem cell differentiation. At the same time, IDH mutations can upregulate vascular endothelial growth factor (VEGF) to promote the formation of the tumor microenvironment. In addition, IDH mutations can also induce high levels of hypoxia-inducible factor-1α (HIF-1α) to promote glioma invasion. Ultimately, these changes will lead to the development of glioma. Currently, a large number of IDH inhibitors and vaccines have entered clinical trials, representing progress in the treatment of glioma patients.

## Introduction

Glioma is the most frequent brain tumor, accounting for more than 60% of primary brain tumors (1). Additionally, gliomas have a notably high mortality and disability rate. For its complex pathogenesis, the existing surgical and drug-assisted treatments are not effective (2). Therefore, it is of great significance to find new targets for diagnosis and treatment. Histologically, the World Health Organization (WHO) classification of tumors of the central nervous system (CNS) distinguishes astrocytomas, oligodendrogliomas, and ependymomas (3, 4). Additionally, gliomas are classified into four grades according to the degree of malignancy by the WHO. Types I and II are low-grade gliomas, and types III and IV are high-grade gliomas (3). However, the revised WHO classification of tumors of the CNS of 2016 combines biology-driven molecular marker diagnostics with classical histological cancer diagnosis (4, 5). Gliomas are reclassified by molecular similarity beyond histological boundaries: diffuse astrocytoma [isocitrate dehydrogenase (IDH) mutant, wild-type, or not otherwise specified (NOS)], gemistocytic astrocytoma (IDH mutant), anaplastic astrocytoma (IDH mutant, wild-type, or NOS), glioblastoma (IDH mutant, wild-type, or NOS), diffuse midline glioma (H3K27M mutant), oligodendroglioma (IDH mutant and 1p/19q codeleted or NOS), anaplastic oligodendroglioma (IDH mutant and 1p/19q codeleted or NOS), oligoastrocytoma (NOS), and anaplastic oligoastrocytoma (NOS) (4, 5). That reclassification improves outcome prediction and will increasingly guide treatment decisions. Tumor evolution of gliomas can be tracked by detecting IDH in cerebrospinal fluid (6). The mutant-IDH1/2 enzyme inhibitors have entered clinical trials for patients with IDH1/2 mutations and represent a novel drug class for targeted therapy.

## IDH

**Figure 1:**
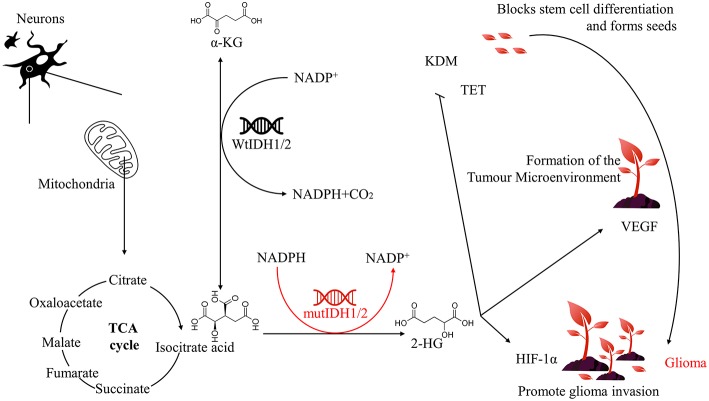
Relationship between IDH1/2 mutation and glioma.

IDH is a small molecule protein that is mainly distributed in the liver, heart muscle and skeletal muscle (7). IDH is involved in a number of cellular processes, including mitochondrial oxidative phosphorylation, glutamine metabolism, lipogenesis, glucose sensing, and regulation of cellular redox status (8, 9). IDH catalyses the oxidative decarboxylation of isocitrate to alpha-ketoglutarate (α-KG) and plays an important role in the reduction of NADP^+^ to reduced nicotinamide adenine dinucleotide phosphate NADPH (Figure 1). IDH has two forms of existence *in vivo*: NADP-dependent IDH (IDH1 and IDH2) and NAD-dependent IDH (IDH3) (10). Among them, IDH1 is present in the cytoplasm and peroxisomes and has antioxidation effects in eukaryotes. Additionally, IDH1 both maintains the antioxidant system *in vivo* and promotes lipid synthesis. While IDH2 is present in mitochondria. This protein plays a key role in tricarboxylic acid (TCA) cycle regulation in multiple tissues and is mainly involved in cellular energy metabolism (11). Currently, IDH1 and IDH2 mutations have been identified in acute myelogenous leukemia, low-grade glioma, and secondary glioblastoma. While IDH3 mutations do not occur at an appreciable frequency in glioblastoma (12). Therefore, we focus exclusively on the roles of IDH1 and IDH2 in glioma biology in this article.

As early as 2008, Parsons et al found a link between glioma and IDH mutation in the exon sequencing of glioblastoma. These researchers also found the IDH1 gene in more than 1/5 of the tumor samples. The arginine (R) at position 132 is converted to histidine (H) (13). Further studies have found that the IDH1 R132H mutation is the most common mutation in gliomas, while the IDH2 gene also undergoes similar mutations at R172, but the frequency of such mutations are relatively low (14). A subsequent range of correlation studies found that IDH mutations were present in 80–90% of grade II and III gliomas (14, 15). The study also found that IDH1/2 mutations are relatively independent and mutually exclusive with few mutations at the same time (16).

## IDH1/2 Mutations and the Development of Glioma

Mutations in genes encoding enzymes of the TCA cycle often contribute to cancer development and progression by disrupting cell metabolism and altering the epigenetic landscape. IDH catalyses the production of α-KG from isocitrate, and when the IDH1/2 gene is mutated, its corresponding function and product will change. This protein inhibits glioma stem cell differentiation by producing high levels of 2-hydroxyglutaric acid (2-HG), upregulates vascular endothelial growth factor (VEGF) to promote tumor microenvironment formation, and produces high levels of hypoxia-inducible factor-1α (HIF-1α) to promote glioma invasion, which ultimately leads to the development of glioma (Figure 1).

## IDH1/2 Mutations and the Differentiation of Glioma Stem Cell

Glioblastoma stem cells refer to a very small number of tumor cells that act as stem cells in glioma cells. According to the “seed and soil” theory put forward by Paget's father Paget (17), if the tumor microenvironment is soil, then glioma stem cells are seeds, which are closely related to the occurrence and invasion of glioma. It was found that 2-HG could compete to occupy the binding position of α-KG due to the high similarity between 2-HG and α-KG; therefore, 2-HG could be regarded as a competitive inhibitor of α-KG-dependent dioxygenase (18). The α-KG-dependent dioxygenase mainly includes histone demethylase KDM and ten-eleven translocation (TET), and its catalytic activity involves many physiological and pathological processes, including angiogenesis, hypoxic stress, and mature differentiation of cells. These processes are closely related to the occurrence and development of tumors (19, 20). IDH1/2 mutations confer a gain of function in glioma cells, resulting in the accumulation and secretion of a vast excess of an oncometabolite, D-2-HG, which ultimately inhibits the catalytic activity of α-KG-dependent dioxygenase, damaging the key steps in histone modification and DNA demethylation (21). This hypermethylation state caused by IDH1/2 mutations are widely present in the CpG island of the human malignant tumor genome. It is noteworthy that such changes are more likely to occur in cancer stem cells of IDH1/2 mutant tumors (19). The CpG islands are not only a marker of genes but are also involved in the regulation of gene expression and the structure of chromatin. Studies have shown that the hypermethylation status of CpG islands leads to the inactivation of tumor suppressor genes and this epigenetic modification. Hypermethylation of CpG islands is associated with altered gene expression involved in cell differentiation (22). Therefore, IDH1/2 mutations block glioma stem cell differentiation (20). However, the study found that 2-HG is a weak competitive inhibitor of α-KG. Therefore, it can only be observed to inhibit the differentiation of glioma stem cells when high levels of 2-HG accumulate (18), and the high levels of 2-HG alone will not cause glioma (23). Furthermore, the development of glioma requires not only seeds (glioma stem cells) but also soil (tumor microenvironment).

## IDH1/2 Mutations and the Tumor Microenvironment

Similar with the way that plants modify their surroundings, tumor seeds can also change the environment in which they exist. By promoting the formation of microvasculature, tumor seeds can acquire growth requirements, produce long-term low inflammation and inhibit immune clearance, which is also an important reason for drug resistance and escape from immune system examination. It was found that the mutations of IDH1/2 could promote the formation of the tumor microenvironment by increasing the expression of VEGF and make it suitable for glioblastoma stem cell development (24). Simultaneously, the expression of VEGF in the glioma group of the same level was higher than that of the non-mutation group (25, 26). It is worth noting that HIF-1α can initiate transcription of VEGF, and hypoxia can induce an increase in VEGF (26). The IDH1/2 mutations can upregulate VEGF to promote tumor microvessel formation by inhibiting the breakdown of HIF-1α (27). The newly formed tumor microenvironment provides nutritional support for glioma cells through exosomes (24), and a large amount of IDH1 mRNA expression is detected in the cerebrospinal fluid of glioma patients (28). The IDH1/2 mutations make the tumor microenvironment easier to form. With the proper soil, the glioma stem cells grow rapidly and continue to invade the surrounding tissues, eventually accelerating the development of glioma.

## IDH1/2 Mutations and Glioma Invasion

The invasiveness of glioma is the main reason for its recurrence. The tumor cells remaining after surgery are more invasive, as plants will accelerate their sexual maturity process under adverse external conditions and then produce the next generation earlier to escape from the bad environment. Continuous seeding of tumor seeds will further promote the development of tumors, and IDH1/2 mutations play an important role in this process. We know that the rapid growth of tumors will quickly consume the surrounding energy and nutrients. Therefore, glioma cells exhibit invasive characteristics after human treatment or in the late stage of the disease, mainly in the pursuit of nutrients and escape from adverse environments. van Lith et al found that IDH2 mutation can induce nuclear accumulation of β-catenin and upregulated HIF-1α, which is closely related to tumor invasion and chemoresistance, manifested as a search for glutamate (29). In addition, the IDH1/2 mutations can cause abnormal expression of platelet-derived growth factor (PDGF). In the PDGF-induced glioma model, tumor cells exhibit notable invasive properties, and this effect can lead to abnormal activation of microglia, further promoting the transformation of tumor cells into migration morphology (30, 31). Both PDGF and HIF-1α are closely related to oxygen, and oxygen is a key component of energy metabolism. Therefore, we hypothesize that invasiveness is the pursuit of energy and escape from the adverse environment of tumor cells. We can further explain the relationship between IDH1/2 mutations and HIF-1α. The HIF-1α is stably expressed under hypoxia, and its expression is mainly caused by prolyl hydroxylase (PHD). The hydroxylation is regulated in a concentration-dependent manner. Notably, PHD is an α-KG-dependent dioxygenase (27).

The IDH1/2 mutations cause α-KG-dependent dioxygenase activity to be inhibited (21). The experimental results show that the expression of HIF-1α is significantly upregulated in tumor cells surrounding the necrotic area of glioma due to exposure to hypoxia, and these cells often exhibit migration patterns (32). This finding indicates that glioma cells are more likely to escape from the hypoxic environment and necrotic areas in the case of IDH1/2 mutations, therefore the glioma cells have stronger invasiveness. Oxygen content is closely related to the distribution of microvessels in addition to the migration of glioma cells, and the microvascular proliferation of tumor abnormalities is closely related to the formation of the tumor microenvironment. Therefore, these changes caused by the IDH1/2 mutations will eventually lead to the development of glioma.

## IDH1/2 Inhibitors and Vaccines

In view of the important role of IDH1/2 mutations in the occurrence and development of glioma, studies of these proteins may enable researchers to identify appropriate inhibitors and intervene in the treatment of glioma patients with IDH1/2 mutations. Many IDH1/2 inhibitors have entered the stage of clinical trials. In addition, vaccines against IDH1/2 mutants have also been studied (Table 1).

**Table 1:** In glioma: clinical trials of IDH1/2 inhibitors, vaccines and new uses for old drugs.

**ClinicalTrials.gov**	**Drug**	**Phase**	**Study title**	**References**
NCT02073994	AG-120	II	Study of AG-120 in subjects with advanced solid tumors, including glioma with an IDH1 mutation	(33)
NCT03343197	AG-120+AG-881	II	Study of AG-120 and AG-881 in subjects with low grade glioma	(34)
NCT02273739	AG-221	I II	Study of orally administered AG-221 in subjects with advanced solid tumors, including glioma, and with angioimmunoblastic T-cell lymphoma, with an IDH2 mutation subjects with advanced solid tumors, including glioma, and with angioimmunoblastic T-cell	(35)
NCT02454634	IDH1 peptide vaccine	I	Phase I trial of IDH1 peptide vaccine in IDH1R132H-mutated grade III-IV gliomas IDH1	(36)
NCT02193347	IDH1peptide vaccine	I	IDH1 peptide vaccine for recurrent grade II glioma (RESIST)	(37)
NCT02771301	IDH1R132H-DC vaccine	I	Safety and efficacy of IDH1R132H-DC vaccine in gliomas	(38)
NCT03666559	Azacitidine	II	Treatment with azacitidine of recurrent gliomas with IDH1/2 mutation	(39)
NCT03557359	Nivolumab	II	Nivolumab for recurrent or progressive IDH mutant gliomas	(40)
NCT02766270	Temozolomide	Early phase I	CCRT with temozolomide vs. RT alone in patients with IDH wild-type/TERT promoter mutation grade II/III gliomas	(41)
NCT02209428	Temozolomide	II	Prospective cohort to study the effect of temozolomide on IDH mutational low-grade gliomas	(42)
NCT03684811	FT-2102+azacitidine	I and II	Study of FT 2102 in participants with advanced solid tumors and gliomas with an IDH1 mutation	(43)

## Inhibitor

IDH1/2-mutant inhibitors have been shown to have good effects in preclinical studies. The inhibitors can both quantitatively inhibit IDH1/2 mutants and reduce 2-HG dose-dependently to normal levels and partially reverse histone modification and DNA hypermethylation, thereby playing a protective role. The AGI-5198 (the first selective IDH1 R132H/R132C mutant inhibitor) reduced 2-HG in a dose-dependent manner and inhibited the growth of tumors both *in vitro* and *in vivo*. In the IDH1-mutant glioma model, AGI-5198 induces the expression of genes related to the differentiation of astrocytes and oligodendrocytes and reduces the inhibitory histones at the promoters of these genes, thereby promoting the differentiation of glioma cells (44). In addition to preclinical studies, IDH1/2 mutants enzyme inhibitors also show great potential in clinical trials. Ivosidenib (AG-120) and enasidenib (AG-221) are the preferred reversible selective inhibitors of IDH1 and IDH2 mutant enzymes, respectively. AG-221, as a therapeutic drug for acute myelocytic leukemia (AML), has been approved by the US FDA on August 1, 2017, and is the first cancer metabolic drug accepted by FDA (45). Interestingly, as early as 2014, a clinical study of oral AG-221 was carried out in patients with advanced solid tumors, including gliomas with IDH2 mutation and angioimmunoblastic T-cell lymphoma. AG-221 showed inhibitory effects on solid tumors, but due to the complexity of the development of these indications, the cycle is considerably longer than that in AML. Therefore, further clinical trials are needed for verification (35). Since the IDH1 R132H mutation is more common in gliomas, more clinical trials have been conducted for IDH1. In 2014, a phase I/II clinical trial (NCT02073994), named “Study on IDH1 mutation of AG-120 in patients with advanced solid tumors including gliomas,” was conducted, which mainly verified the safety and tolerance of AG-120 and preliminarily explored the clinical therapeutic effect of AG-120 (33, 46). Recently, Agios Pharmaceutical has conducted a clinical study, called “AG-120 and Vorasidenib (AG-881) in patients with low-grade glioma” (NCT03343197) (34). This study is a phase I multicentre clinical study of recurrent non-enhanced gliomas. Patients requiring surgery have mutation in IDH1 R132H. The aim of this study was to evaluate the inhibition of 2-HG by comparing 2-HG concentrations in excised and untreated control tumors of IDH1 mutant glioma subjects treated with AG-120 or AG-881 (non-specific IDH inhibitors). Data on clinical safety, tolerance, pharmacokinetics/pharmacodynamics (PK/PD), and antineoplastic activity of subjects with IDH1 R132H mutation in recurrent non-enhanced low-grade glioma will be studied. This study will provide recommended doses of AG-120 and AG-881 for future glioma research. The early data on AG-120 as a tumor metabolic regulator in clinical trials are similar to the efficacy and side effects of AG-221 already on the market (33, 45, 46). Since IDH1/2 mutants enzyme inhibitors have good application prospects in pre-clinical research and clinical trials of glioma, we believe that IDH1/IDH2 mutant enzyme inhibitors will bring new hope to glioma patients.

## Vaccines

Vaccination is the most effective method of disease prevention and control. Many viruses and bacteria that once caused catastrophic pandemics (e.g., smallpox, poliomyelitis, measles, and diphtheria) are either eradicated or effectively controlled through routine vaccination programmes. Therefore, is the development of the IDH1/2 vaccine conducive to the treatment of glioma? The answer is obvious. In some low-grade glioma patients, the spontaneous immune response to IDH1 mutation has been found (47). The use of the self-immune response to treat tumors has also been a heavily researched subject in recent years. Therefore, immunization therapy may be a new hope for the treatment of glioma. The IDH1 R132H mutation occurs in 70% of low-grade gliomas (48). The researchers used 15 amino acids to construct artificial IDH1 polypeptides with this characteristic mutation and injected mice with human major histocompatibility complex (MHC) molecules with these artificial polypeptides to produce specific vaccines. In animal experiments, it was found that IDH1 mutant cancer cells could be prevented from growing in the brain, and the vaccine did not destroy the normal physiological function of the IDH1 enzyme (47). In addition, by site-directed mutagenesis, the R132H mutation was introduced into the mouse glioma cell line GL261 to produce the mIDH1-GL261 cell line. Mice with p-GL261 glioma treated with mIDH1-GL261 survived longer than the control group, even 25% of them were cured. Immunized mice showed a higher number of peripheral CD8^+^ T cells, higher levels of IFN-gamma and anti-mIDH1 antibodies (49). These results indicate the potential of the vaccine in the treatment of glioma patients with IDH1 mutation. Subsequently, the German National Cancer Center launched a Phase I trial, IDH1 Peptide Vaccine in IDH1 R132H Mutant III-IV Glioma (NOA-16) (NCT02454634) (36), and Duke University also launched a clinical trial, called “IDH1 Peptide Vaccine for Recurrent Grade II Glioma (RESIST)” (NCT02193347) (37). In addition, The Tiantan Hospital and Yanda International Hospital in China also launched a clinical trial of “Safety and Effectiveness of IDH1 R132H-DC Vaccine in Glioma” (NCT02771301) (38).

It is difficult for gliomas to be completely cleared by surgery and drugs, so they often recur, and the recurrent gliomas after clearance generally have stronger resistance and invasiveness. Vaccines can play a sustained role in patients. Therefore, finding a suitable IDH1/2 mutations vaccine will benefit patients greatly and help them escape the magic spell of glioma recurrence.

## New Uses for Old Drugs

It is generally known that trials of IDH mutations inhibitors and vaccines in IDH mutant gliomas and recurrent gliomas have been conducted. Meanwhile, old drugs for other tumors have also been developed to treat glioma with IDH1/2 mutations (Table 1), such as azacitidine, nivolumab, and temozolomide.

Preclinical data have shown a dramatic anti-tumor effect of hypomethylating drugs, such as 5-azacytidine, on IDH1-mutated human gliomas. These hypomethylating drugs are routinely used in myelodysplastic syndrome (MDS) and are well-tolerated. A clinical trial treating recurrent gliomas with IDH1/2 mutations with azacitidine was conducted. The main objective is to evaluate the efficacy of azacytidine according to the response assessment in neuro-oncology (RANO) criteria on progression-free survival at 6 months, evaluated according to the RANO criteria (39). Additionally, a Phase II, open-label, single-arm study of nivolumab for recurrent or progressive IDH mutant gliomas with prior exposure to alkylating agents also was conducted. The study aimed to determine the response rates to nivolumab of recurrent or progressive IDH mutations high-grade gliomas with prior exposure to alkylating agents (40). Jinsong Wu's study provides a higher level (IIb) of evidence for the correlation between IDH mutations and the responsiveness to up-front adjuvant metronomic temozolomide chemotherapy in young patients with low grade gliomas located in eloquent brain areas (42). The clinical trials implemented by Tao Jiang also support temozolomide as an adjuvant therapy for lower-grade glioma (41). Moreover, the current Phase 1/2 study evaluated the safety, efficacy, PK, and PD of Olutasidenib (FT-2102, which is a selective potent inhibitor of IDH1) as a single agent and in combination with other anticancer drugs in patients with advanced solid tumors and gliomas (43). We hope that these clinical trials will be successful and will bring hope to patients.

## Conclusions

The IDH1/2 mutations can produce a high level of 2-HG to inhibit the differentiation of glioma stem cells, upregulate the formation of the tumor microenvironment, and produce a high level of HIF-1α to promote the invasion of glioma. Ultimately, these changes will lead to the occurrence and development of glioma (Figure 1). Therefore, IDH1/2 is an important target for the prevention and treatment of glioma. However, due to the long and uncertain clinical trial cycle times, most of the clinical trials of IDH1/2 inhibitors and vaccines are still in progress. There is still considerable research to be accomplished. However, we believe that the great potential of IDH1/2 inhibitors and vaccines will bring hope to glioma patients.

## Author Contributions

All authors listed have made a substantial, direct and intellectual contribution to the work, and approved it for publication.

### Conflict of Interest Statement

The authors declare that the research was conducted in the absence of any commercial or financial relationships that could be construed as a potential conflict of interest.

## References

[1] Ostrom QT Gittleman H Liao P Vecchione-Koval T Wolinsky Y Kruchko C. CBTRUS Statistical Report: primary brain and other central nervous system tumors diagnosed in the United States in 2010–2014. Neuro Oncol. (2017) 19:v1–88. 10.1093/neuonc/nox158 29117289 PMC5693142

[2] Ohba S Mukherjee J See WL Pieper RO. Mutant IDH1-driven cellular transformation increases RAD51-mediated homologous recombination and temozolomide resistance. Cancer Res. (2014) 74:4836–44. 10.1158/0008-5472.CAN-14-0924 25035396 PMC4154998

[3] Louis DN Ohgaki H Wiestler OD Cavenee WK Burger PC Jouvet A. The 2007 WHO classification of tumours of the central nervous system. Acta Neuropathol. (2007) 114:97–109. 10.1007/s00401-007-0243-4 17618441 PMC1929165

[4] Louis DN Perry A Reifenberger G von Deimling A Figarella-Branger D Cavenee WK. The 2016 World Health Organization classification of tumors of the central nervous system: a summary. Acta Neuropathol. (2016) 131:803–20. 10.1007/s00401-016-1545-1 27157931

[5] Wirsching HG Weller M. The role of molecular diagnostics in the management of patients with gliomas. Curr Treat Options Oncol. (2016) 17:51. 10.1007/s11864-016-0430-4 27501915

[6] Miller AM Shah RH Pentsova EI Pourmaleki M Briggs S Distefano N. Tracking tumour evolution in glioma through liquid biopsies of cerebrospinal fluid. Nature. (2019) 565:654–8. 10.1038/s41586-019-0882-3 30675060 PMC6457907

[7] LaPorte DC Koshland DE Jr. Phosphorylation of isocitrate dehydrogenase as a demonstration of enhanced sensitivity in covalent regulation. Nature. (1983) 305:286–90. 10.1038/305286a0 6312317

[8] Reitman ZJ Yan H. Isocitrate dehydrogenase 1 and 2 mutations in cancer: alterations at a crossroads of cellular metabolism. J Natl Cancer Inst. (2010) 102:932–41. 10.1093/jnci/djq187 20513808 PMC2897878

[9] Stander M Peraud A Leroch B Kreth FW. Prognostic impact of TP53 mutation status for adult patients with supratentorial World Health Organization Grade II astrocytoma or oligoastrocytoma: a long-term analysis. Cancer. (2004) 101:1028–35. 10.1002/cncr.20432 15329912

[10] Yen KE Bittinger MA Su SM Fantin VR. Cancer-associated IDH mutations: biomarker and therapeutic opportunities. Oncogene. (2010) 29:6409–17. 10.1038/onc.2010.444 20972461

[11] Waitkus MS Diplas BH Yan H. Isocitrate dehydrogenase mutations in gliomas. Neuro Oncol. (2016) 18:16–26. 10.1093/neuonc/nov136 26188014 PMC4677412

[12] Krell D Assoku M Galloway M Mulholland P Tomlinson I Bardella C. Screen for IDH1, IDH2, IDH3, D2HGDH and L2HGDH mutations in glioblastoma. PLoS ONE. (2011) 6:e19868. 10.1371/journal.pone.0019868 21625441 PMC3100313

[13] Parsons DW Jones S Zhang X Lin JC Leary RJ Angenendt P. An integrated genomic analysis of human glioblastoma multiforme. Science. (2008) 321:1807–12. 10.1126/science.1164382 18772396 PMC2820389

[14] Hartmann C Meyer J Balss J Capper D Mueller W Christians A. Type and frequency of IDH1 and IDH2 mutations are related to astrocytic and oligodendroglial differentiation and age: a study of 1,010 diffuse gliomas. Acta Neuropathol. (2009) 118:469–74. 10.1007/s00401-009-0561-9 19554337

[15] Cohen AL Holmen SL Colman H. IDH1 and IDH2 mutations in gliomas. Curr Neurol Neurosci Rep. (2013) 13:345. 10.1007/s11910-013-0345-4 23532369 PMC4109985

[16] Kloosterhof NK Bralten LB Dubbink HJ French PJ van den Bent MJ. Isocitrate dehydrogenase-1 mutations: a fundamentally new understanding of diffuse glioma? Lancet Oncol. (2011) 12:83–91. 10.1016/S1470-2045(10)70053-X 20615753

[17] DeVita VT Jr. Rosenberg SA. Two hundred years of cancer research. N Engl J Med. (2012) 366:2207–14. 10.1056/NEJMra1204479 22646510 PMC6293471

[18] Xu W Yang H Liu Y Yang Y Wang P Kim SH. Oncometabolite 2-hydroxyglutarate is a competitive inhibitor of alpha-ketoglutarate-dependent dioxygenases. Cancer Cell. (2011) 19:17–30. 10.1016/j.ccr.2010.12.014 21251613 PMC3229304

[19] Figueroa ME Abdel-Wahab O Lu C Ward PS Patel J Shih A. Leukemic IDH1 and IDH2 mutations result in a hypermethylation phenotype, disrupt TET2 function, and impair hematopoietic differentiation. Cancer Cell. (2010) 18:553–67. 10.1016/j.ccr.2010.11.015 21130701 PMC4105845

[20] Lu C Ward PS Kapoor GS Rohle D Turcan S Abdel-Wahab O. IDH mutation impairs histone demethylation and results in a block to cell differentiation. Nature. (2012) 483:474–8. 10.1038/nature10860 22343901 PMC3478770

[21] Dang L White DW Gross S Bennett BD Bittinger MA Driggers EM. Cancer-associated IDH1 mutations produce 2-hydroxyglutarate. Nature. (2009) 462:739–44. 10.1038/nature08617 19935646 PMC2818760

[22] Doi A Park IH Wen B Murakami P Aryee MJ Irizarry R. Differential methylation of tissue- and cancer-specific CpG island shores distinguishes human induced pluripotent stem cells, embryonic stem cells and fibroblasts. Nat Genet. (2009) 41:1350–3. 10.1038/ng.471 19881528 PMC2958040

[23] Kranendijk M Struys EA van Schaftingen E Gibson KM Kanhai WA van der Knaap MS. IDH2 mutations in patients with D-2-hydroxyglutaric aciduria. Science. (2010) 330:336. 10.1126/science.1192632 20847235

[24] Zhao H Yang L Baddour J Achreja A Bernard V Moss T. Tumor microenvironment derived exosomes pleiotropically modulate cancer cell metabolism. Elife. (2016) 5:e10250. 10.7554/eLife.10250 26920219 PMC4841778

[25] Veganzones S de la Orden V Requejo L Mediero B Gonzalez ML Del Prado N. Genetic alterations of IDH1 and Vegf in brain tumors. Brain Behav. (2017) 7:e00718. 10.1002/brb3.718 28948065 PMC5607534

[26] Yalaza C Ak H Cagli MS Ozgiray E Atay S Aydin HH. R132H mutation in IDH1 gene is associated with increased tumor HIF1-alpha and serum VEGF levels in primary glioblastoma multiforme. Ann Clin Lab Sci. (2017) 47:362–4. 28667042

[27] Pientka FK Hu J Schindler SG Brix B Thiel A Johren O. Oxygen sensing by the prolyl-4-hydroxylase PHD2 within the nuclear compartment and the influence of compartmentalisation on HIF-1 signalling. J Cell Sci. (2012) 125:5168–76. 10.1242/jcs.109041 22946054

[28] Chen WW Balaj L Liau LM Samuels ML Kotsopoulos SK Maguire CA. BEAMing and droplet digital PCR analysis of mutant IDH1 mRNA in glioma patient serum and cerebrospinal fluid extracellular vesicles. Mol Ther Nucleic Acids. (2013) 2:e109. 10.1038/mtna.2013.28 23881452 PMC3732870

[29] van Lith SA Molenaar R van Noorden CJ Leenders WP. Tumor cells in search for glutamate: an alternative explanation for increased invasiveness of IDH1 mutant gliomas. Neuro Oncol. (2014) 16:1669–70. 10.1093/neuonc/nou152 25074540 PMC4232089

[30] Flavahan WA Drier Y Liau BB Gillespie SM Venteicher AS Stemmer-Rachamimov AO. Insulator dysfunction and oncogene activation in IDH mutant gliomas. Nature. (2016) 529:110–4. 10.1038/nature16490 26700815 PMC4831574

[31] Masui K Kato Y Sawada T Mischel PS Shibata N. Molecular and genetic determinants of glioma cell invasion. Int J Mol Sci. (2017) 18:E2609. 10.3390/ijms18122609 29207533 PMC5751212

[32] Bello L Giussani C Carrabba G Pluderi M Costa F Bikfalvi A. Angiogenesis and invasion in gliomas. Cancer Treat Res. (2004) 117:263–84. 10.1007/978-1-4419-8871-3_16 15015565

[33] Study of Orally Administered AG-120 in Subjects With Advanced Solid Tumors, Including Glioma, With an IDH1 Mutation. (2014). Available online at: https://clinicaltrials.gov/show/NCT02073994 (accessed February 28, 2014).

[34] Study of AG-120 and AG-881 in Subjects With low Grade Glioma. (2017). Available online at: https://clinicaltrials.gov/show/NCT03343197 (accessed November 17, 2017).

[35] Study of Orally Administered AG-221 in Subjects With Advanced Solid Tumors, Including Glioma, and With Angioimmunoblastic T-cell Lymphoma, With an IDH2 Mutation Subjects With Advanced Solid Tumors, Including Glioma, and With Angioimmunoblastic T-cell Lymphoma, With an IDH2 Mutation. (2014). Available online at: https://clinicaltrials.gov/show/NCT02273739 (accessed October 24, 2014).

[36] Phase I Trial of IDH1 Peptide Vaccine in IDH1R132H-Mutated Grade III-IV Gliomas. (NOA-16). (2015). Available online at: https://clinicaltrials.gov/show/NCT02454634 (accessed May 27, 2015).

[37] IDH1 Peptide Vaccine for Recurrent Grade II Glioma. (RESIST). (2014). Available online at: https://clinicaltrials.gov/show/NCT02454634 (accessed July 17, 2014).

[38] Safety and Efficacy of IDH1R132H-DC Vaccine in Gliomas. (2016). Available online at: https://clinicaltrials.gov/show/NCT02771301 (accessed May 13, 2016).

[39] Treatment With Azacitidine of Recurrent Gliomas With IDH1/2 Mutation. (AGIR). (2018). Available online at: https://www.clinicaltrials.gov/show/NCT03666559 (accessed September 12, 2018).

[40] Nivolumab for Recurrent or Progressive IDH Mutant Gliomas. (2018). Available online at: https://www.clinicaltrials.gov/ct2/show/NCT03557359 (accessed June 15, 2018).

[41] CCRT With Temozolomide Versus RT Alone in Patients With IDH Wild-Type/TERT Promoter Mutation Grade II/III Gliomas. (2016). Available online at: https://www.clinicaltrials.gov/ct2/show/NCT02766270 (accessed May 13, 2016).

[42] A Prospective Cohort to Study the Effect of Temozolomide on IDH Mutational low Grade Gliomas. (2014). Available online at: https://www.clinicaltrials.gov/ct2/show/NCT02209428 (accessed August 5, 2014).

[43] A Study of FT 2102 in Participants With Advanced Solid Tumors and Gliomas With an IDH1 Mutation. (2018). Available online at: https://www.clinicaltrials.gov/ct2/show/NCT03684811 (accessed September 26, 2018).

[44] Rohle D Popovici-Muller J Palaskas N Turcan S Grommes C Campos C. An inhibitor of mutant IDH1 delays growth and promotes differentiation of glioma cells. Science. (2013) 340:626–30. 10.1126/science.1236062 23558169 PMC3985613

[45] FDA Granted Regular Approval to Enasidenib for the Treatment of Relapsed or Refractory AML. (2017). Available online at: https://www.fda.gov/drugs/informationondrugs/approveddrugs/ucm569482.htm

[46] Fan B Le K Manyak E Liu H Prahl M Bowden CJ Longitudinal pharmacokinetic/pharmacodynamic profile of AG-120, a potent inhibitor of the IDH1 mutant protein, in a phase 1 study of IDH1-mutant advanced hematologic malignancies. Am. Soc. Hematol. (2015) 126:1310 Available online at: http://www.bloodjournal.org/content/126/23/1310

[47] Schumacher T Bunse L Pusch S Sahm F Wiestler B Quandt J. A vaccine targeting mutant IDH1 induces antitumour immunity. Nature. (2014) 512:324–7. 10.1038/nature13387 25043048

[48] Capper D Zentgraf H Balss J Hartmann C von Deimling A. Monoclonal antibody specific for IDH1 R132H mutation. Acta Neuropathol. (2009) 118:599–601. 10.1007/s00401-009-0595-z 19798509

[49] Pellegatta S Valletta L Corbetta C Patane M Zucca I Riccardi Sirtori F. Effective immuno-targeting of the IDH1 mutation R132H in a murine model of intracranial glioma. Acta Neuropathol Commun. (2015) 3:4. 10.1186/s40478-014-0180-0 25849072 PMC4359524

